# Clinical consequences of *BRCA2* hypomorphism

**DOI:** 10.1038/s41523-021-00322-9

**Published:** 2021-09-09

**Authors:** Laia Castells-Roca, Sara Gutiérrez-Enríquez, Sandra Bonache, Massimo Bogliolo, Estela Carrasco, Miriam Aza-Carmona, Gemma Montalban, Núria Muñoz-Subirana, Roser Pujol, Cristina Cruz, Alba Llop-Guevara, María J. Ramírez, Cristina Saura, Adriana Lasa, Violeta Serra, Orland Diez, Judith Balmaña, Jordi Surrallés

**Affiliations:** 1 Genome Instability and DNA repair Syndromes Group and Join Unit UAB-IR Sant Pau on Genomic Medicine, Biomedical Research Institute IIB-Sant Pau, Hospital de la Santa Creu i Sant Pau, Barcelona, Spain; 2 Genetics Department, Hospital de la Santa Creu i Sant Pau, Barcelona, Spain; 3 Hereditary Cancer Genetics Group, Vall d’Hebron Institute of Oncology (VHIO), Vall d’Hebron Barcelona Hospital Campus, Barcelona, Spain; 4 Center for Biomedical Network Research on Rare Diseases (CIBERER) U-745, Barcelona, Spain; 5 Hereditary Cancer Genetics Group, Vall d’Hebron Institute of Oncology (VHIO), Hospital Universitari Vall d’Hebron, Vall d’Hebron Barcelona Hospital Campus, Barcelona, Spain; 6 Experimental Therapeutics Group, Vall d’Hebron Institute of Oncology (VHIO), Vall d’Hebron Barcelona Hospital Campus, Barcelona, Spain; 7 Breast Cancer and Melanoma Group, Vall d’Hebron Institute of Oncology (VHIO), Hospital Universitari Vall d’Hebron, Vall d’Hebron Barcelona Hospital Campus, Barcelona, Spain; 8 Center for Biomedical Network Research on Rare Diseases (CIBERER) U-705, Barcelona, Spain; 9 Present Address: CHU de Québec – Université Laval Research Center, Oncology division, 9 Rue McMahon, Québec city, G1R 3S3 Québec Canada

**Keywords:** Cancer genetics, Breast cancer

## Abstract

The tumor suppressor *FANCD1/BRCA2* is crucial for DNA homologous recombination repair (HRR). *BRCA2* biallelic pathogenic variants result in a severe form of Fanconi anemia (FA) syndrome, whereas monoallelic pathogenic variants cause mainly hereditary breast and ovarian cancer predisposition. For decades, the co-occurrence in *trans* with a clearly pathogenic variant led to assume that the other allele was benign. However, here we show a patient with biallelic *BRCA2* (c.1813dup and c.7796 A > G) diagnosed at age 33 with FA after a hypertoxic reaction to chemotherapy during breast cancer treatment. After DNA damage, patient cells displayed intermediate chromosome fragility, reduced survival, cell cycle defects, and significantly decreased RAD51 foci formation. With a newly developed cell-based flow cytometric assay, we measured single *BRCA2* allele contributions to HRR, and found that expression of the missense allele in a *BRCA2* KO cellular background partially recovered HRR activity. Our data suggest that a hypomorphic *BRCA2* allele retaining 37–54% of normal HRR function can prevent FA clinical phenotype, but not the early onset of breast cancer and severe hypersensitivity to chemotherapy.

## Introduction

Fanconi anemia (FA) is a rare genetic DNA repair syndrome characterized by bone marrow failure (BMF) and cancer predisposition particularly to acute myeloid leukemia (AML) and/or solid tumors, mainly head and neck squamous cell carcinoma (HNSCC)^1^. FA cells exhibit chromosomal instability and hypersensitivity to DNA damage especially to DNA interstrand crosslinks (ICLs)^2^. Twenty-two FA-related genes have been identified including tumor suppressors *BRCA1* (*FANCS*) and *BRCA2* (*FANCD1*).

The breast cancer susceptibility 2 (BRCA2) protein is a key component of the DNA homologous recombination repair (HRR) pathway^3^ and a downstream component of the FA/BRCA DNA repair pathway^1,2^. Monoallelic germline pathogenic variants in *BRCA2* are a common cause of hereditary breast and ovarian cancer (HBOC)^4^, while biallelic pathogenic variants are associated with FA^5^. Individuals with biallelic *BRCA2* pathogenic variants usually suffer from a severe form of FA characterized by BMF, multiple congenital defects, and severe cancer predisposition as patients develop leukemia and/or multiple solid tumors (mainly Wilms and brain tumors) in the first decade of life. The overall risk of cancer in these patients is about 97% by the age of 5 years^5–9^.

Here, we describe an unusual clinical case consisting of a female patient who reached adulthood with no clear suspicion of a defined pathology, despite presenting two germline *BRCA2* variants: a null frameshift variant c.1813dup, p.(Ile605Asnfs*11) and a missense variant c.7796 A > G, p.(Glu2599Gly). Using a *BRCA2* KO HEK293T cell line stably transfected with an HRR GFP marker construct^10^ we demonstrated that the missense allele is partially functional providing 37–54% of HRR activity. We propose that p.(Glu2599Gly) is a hypomorphic allele that provides sufficient HRR activity to prevent FA onset, but it is insufficient to avoid tumorigenesis during adulthood and results in acute sensitivity to chemotherapy.

## Results

### Biallelic *BRCA2*-FA disorder diagnosis

**Fig. 1:**
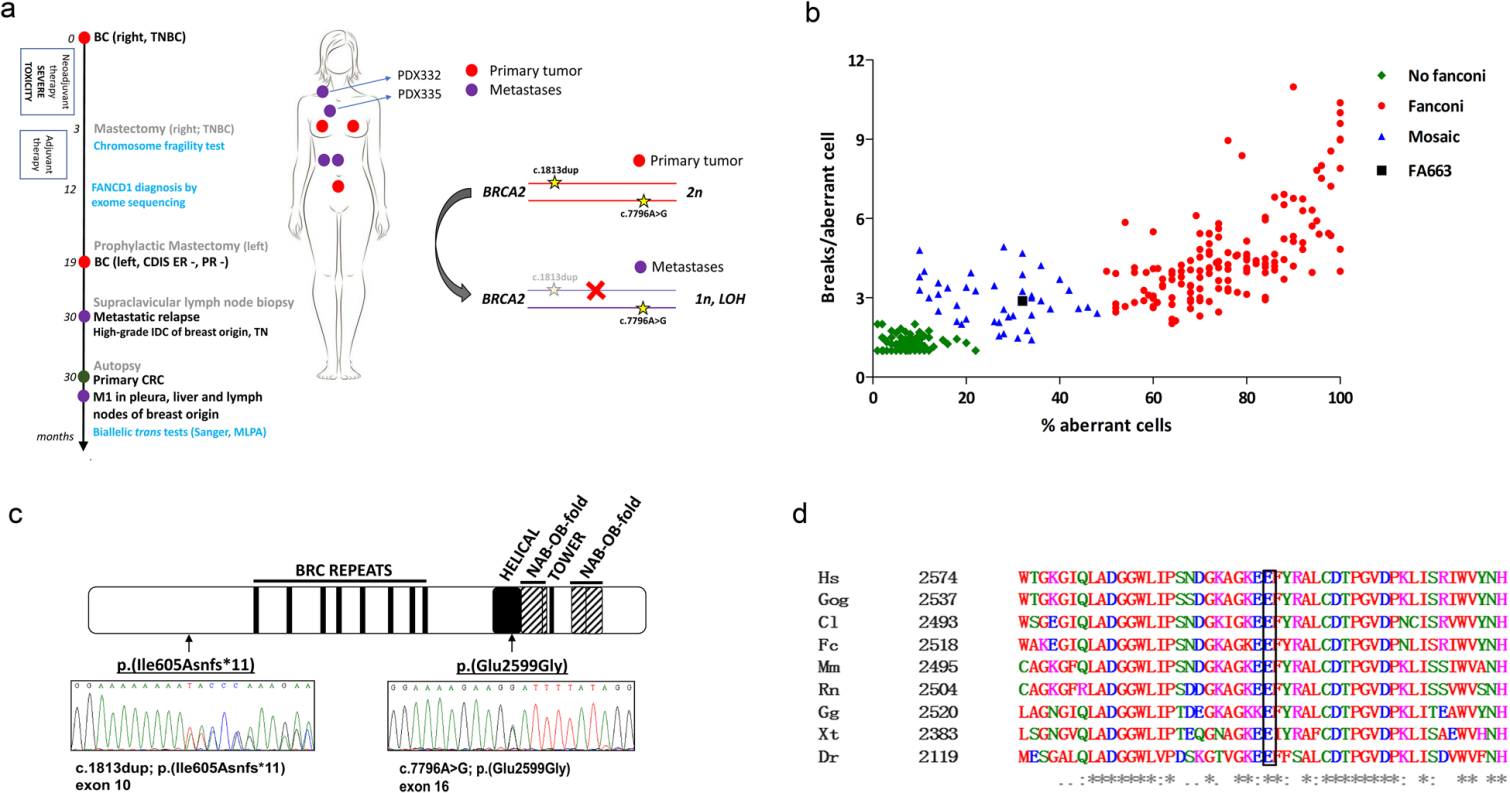
Breast cancer FA663 patient diagnosed with biallelic *BRCA2*-FA disorder. **a** Timeline of patient’s cancer history (diagnoses, treatments, studies) and *BRCA2* analysis. **b** Diepoxybutane (DEB) induced chromosome fragility test (CFT) with blood T-lymphocytes. Percentage distribution of aberrant cells and mean the number of DNA breaks per cell, after DEB induction (0.1 µg/ml). A black square-shaped symbol shows the relative position of FA663 in the distribution of historical data from our laboratory. In the “no Fanconi” group only individuals with at least one aberrant cell have been included. **c** The two *BRCA2* pathogenic variants of FA663. BRCA2 protein (P51587) and InterPro domains schematic drawing. Sequenced loss of function variants found in heterozygosis depicted. **d** the highly conserved amino acid at position 2,599 of the BRCA2 protein. Partial alignment of human BRCA2 with homologous BRCA2 helical domains (IPR015252). BC breast cancer, TNBC triple-negative breast cancer, DCIS ductal carcinoma in situ, IDC invasive ductal carcinoma, ER− estrogen receptors negative, PR− progesterone receptors negative, TN triple-negative, CRC colorectal cancer, M1 metastasis stage 1, MLPA multiplex ligation-dependent probe assay.

Patient FA663 was a thirty-three-year-old woman who developed a malignant breast nodule and, after surgery, showed a severe intolerance to chemotherapy. A timeline of patient’s progression is shown in Fig. 1A. FA663 was an adopted child and her medical history only indicated slight abnormalities such as low birth weight, short stature, a subtle learning disability, skin hyperpigmentation, macrocytosis, hiatal hernia, hepatic steatosis, and lumbar disc herniation. Due to the severe pancytopenia reaction to chemotherapy, a chromosome fragility test (CFT)^11^ was performed in blood T-lymphocytes and the levels of chromosome fragility were compatible with a diagnosis of FA with somatic mosaicism (FA-mosaic) as only one-third of her cells presented multiple chromatid-type breaks and radial figures. (Fig. 1B)^11,12^, FA663 case was then included in a cohort of FA patients to be molecularly characterized by Exome Sequencing (ES)^13^, and two variants were identified in the *BRCA2/FANCD1* gene. The first, c.1813dup, p.(Ile605Asnfs*11) was a clearly pathogenic variant in *BRCA2* exon 10 that produced a frameshift and premature termination of translation (Fig. 1C). This variant had previously been described in FA patients^8^ and in HBOC patients^14^ and it is reported in ClinVar as pathogenic class 5 classified by the ENIGMA expert panel (https://www.ncbi.nlm.nih.gov/clinvar/variation/37762/). A second missense variant c.7796 A > G, p.(Glu2599Gly) was identified in *BRCA2* exon 16 (Fig. 1C) and classified as of “uncertain significance” according to the ACMG guidelines^15^. In ClinVar three submitters also interpreted this variant as of uncertain significance (https://www.ncbi.nlm.nih.gov/clinvar/variation/483130/). Human Glu2599 is a highly conserved residue among organisms in the *BRCA2* DNA binding domain **(**Fig. 1D**)** and the detected variant produced a non-conservative change from a negatively charged amino acid (Glu) to a non-polar amino acid (Gly). The p.(Glu2599Gly) is predicted deleterious for BRASS NN (score 0.912 pathogenic), BRASS MLR (score 1.675 pathogenic), AGVGD (Class C65), PolyPhen-2 (score 1 probably damaging) and SIFT (score 0.01 predict non-tolerated) *in silico* tools. No other potentially pathogenic variants were identified in any other known FA gene.

### Patient fibroblasts retain partial DNA damage response

**Fig. 2:**
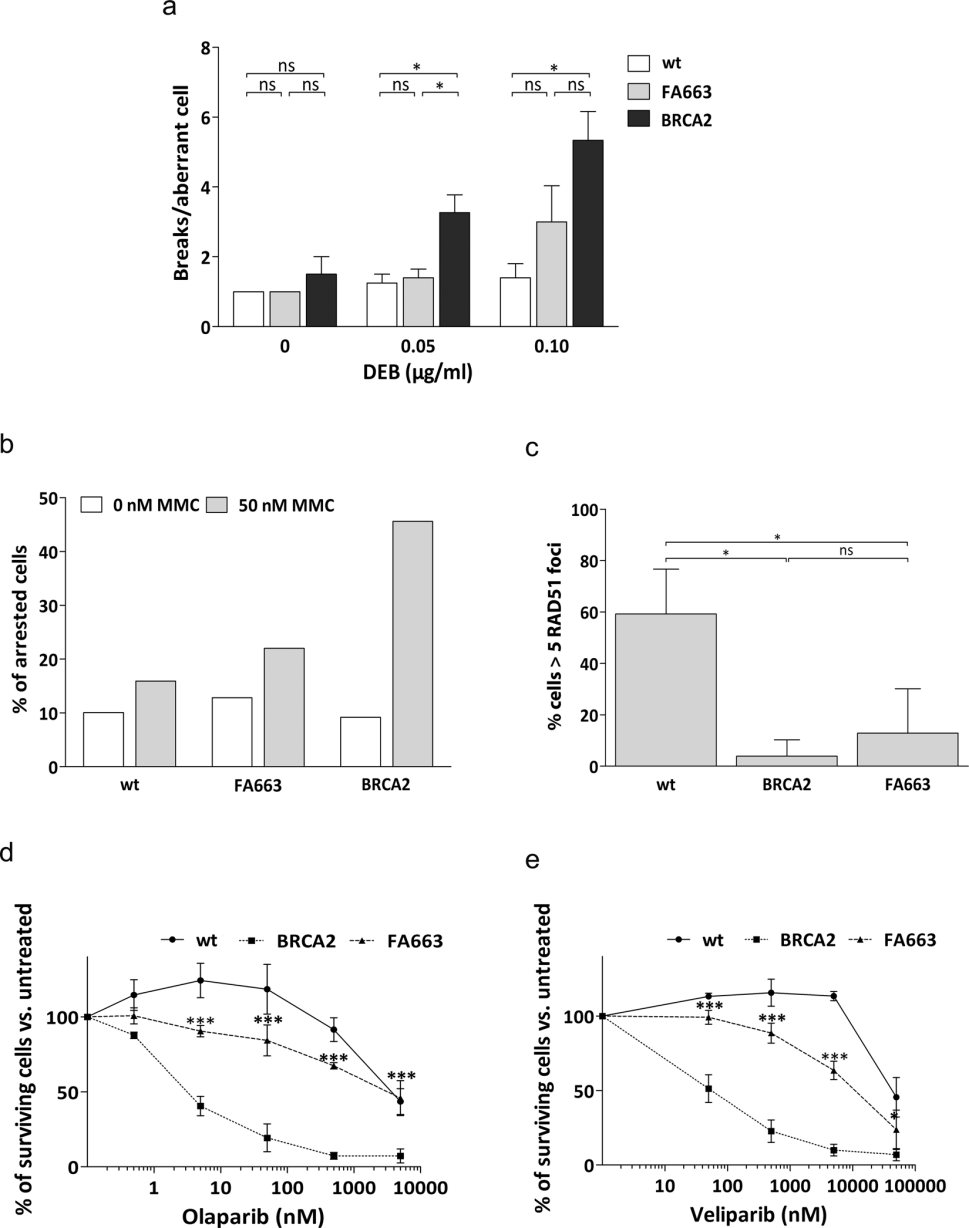
FA663 primary fibroblasts display partial BRCA2 activity. **a** Increased DNA breaks per aberrant cell in FA663 fibroblasts, induced by DEB. The graph represents the mean of 25 aberrant metaphases. Error bars show s.e.m. Statistics are done with a non-parametric Mann-Whitney test, *P < 0.05. **b** MMC induces G_2_/M cell cycle partial arrest in FA663. Cell cycle distribution was monitored by flow cytometry, 72 h after treatment. The representative experiment shown, from three independent data sets. **c** Attenuated RAD51 foci formation in FA663 after IR. Immunofluorescence analysis of wt, *BRCA2*-mutant, and FA663 primary cell lines 6 h after 5 Gy. The graph represents the mean of three independent experiments with duplicates. Error bars show s.e.m. Statistics are done with the One-Way ANOVA test and Bonferroni correction **P < 0.01, ***P < 0.001. **d**, **e** PARPi sensitivity of FA663 primary cells. Data represent the mean of three independent experiments. Error bars represent s.d. Statistics with two-tailed *t*-Test *P < 0.05, ***P < 0.001, comparing BRCA2-mutant cell line. (wt: human primary skin fibroblasts from a healthy individual; BRCA2: primary skin fibroblasts from a homozygous c.469 A > T, p.Lys157* FA patient; FA663: primary skin fibroblasts established from skin biopsy of patient FA663).

To confirm the diagnosis of FA663 as a FA mosaic^11,16^, and to study more in-depth patient’s DNA damage response (DDR), FA663 primary fibroblasts were analyzed together with fibroblasts from a wt control and from a patient with a homozygous *BRCA2* null variant c.469 A > T; p.(Lys157*). In the CFT, FA663 fibroblasts showed a chromosome fragility level in-between the wt and *BRCA2* deficient control and similar to that of blood T-lymphocytes (≈32% aberrant cells; ≈2.88 breaks per aberrant cell) (Fig. 2A). Similarly, intermediate results were also obtained in ICL induced G_2_/M phase cell cycle arrest (Fig. 2B), RAD51 foci formation (Fig. 2C), and sensitivity to the PARP inhibitors olaparib and veliparib (Fig. 2D, E).

### Evidencing the *in trans* position of *BRCA2* c.1813dup and c.7796 A > G variants

**Fig. 3:**
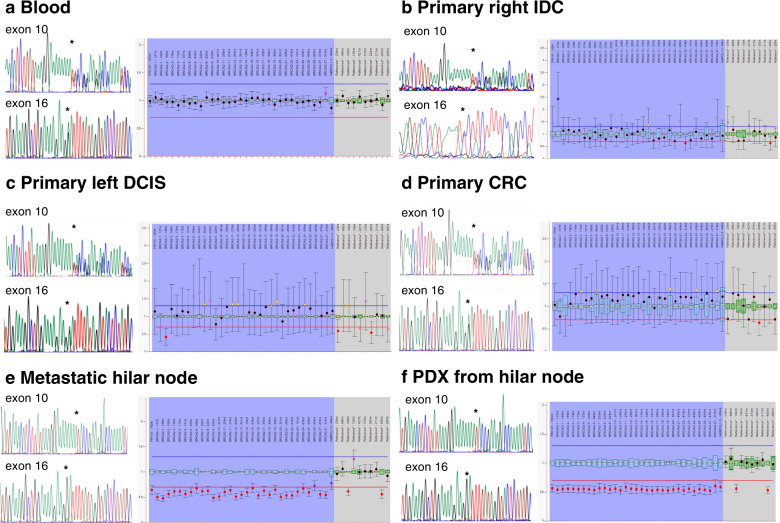
Sanger sequencing and MLPA results from normal and tumor samples indicate that the c.1813dup and c.7796 A > G *BRCA2* variants are in *trans*. Sanger electropherograms for both variants c.1813dup (exon 10) and c.7796 A > G (exon 16) show forward sequences, except for the invasive ductal carcinoma sample (IDC) (**b**) exon 16. MLPA results show the allelic dosage in a ratio chart format as displayed by Coffalyser.Net software. Probe ratios between 0.7 and 1.3 (blue and red lines) showed the presence of two *BRCA2* alleles for blood (**a**), primary IDC (**b**), primary ductal carcinoma in situ (DCIS) (**c**), and primary colorectal cancer (CRC) (**d**). However, probe ratios for metastatic tumors (**e**) and their corresponding PDX **(f**) were around 0.5, suggesting the presence of only one *BRCA2* allele. All Sanger sequences are consistent with their corresponding MLPA findings, i.e. presence of heterozygote variants in Sanger matches with the presence of two allele MLPA evidence while the monoallelic presence of a variant matches with one allele from MLPA outcome. MLPA and Sanger sequences for the skin biopsy and the other metastatic and PDXs samples are shown in Figure S1. *indicates the variant location.

To assess if c.1813dup and c.7796 A > G variants were *in trans*, multiplex ligation-dependent probe assay (MLPA) and Sanger sequencing of the *BRCA2* gene were undertaken in normal tissue samples and tumor samples from FA663 (Table 1). The two allelic variants of *BRCA2* were found in blood, skin tissue, and in three primary tumor samples: invasive ductal carcinoma (IDC), contralateral ductal carcinoma in situ (DCIS), and colorectal cancer (CRC) identified during a necropsy study (Table 1, Fig. 3A–D and Figure S1A). However, four metastatic breast cancer samples (hepatic metastasis and supraclavicular, pleural, hilar, and lymph nodes) infiltrated by the primary breast IDC tumor, and two patient-derived tumor xenograft (PDX) models (derived from supraclavicular and hilar metastases) showed loss of heterozygosity (LOH) in *BRCA2* (Table 1, Fig. 3E–F and Figure S1B–E). Specifically, all the infiltrated nodes and PDXs samples presented exclusively the c.7796 G allele, suggesting that the two variants were located in *trans* (Table 1). In addition, the cDNA obtained from blood RNA and cultured fibroblasts was sequenced by Sanger (Figure S2). We observed a lower signal of the allele carrying the duplication in exon 10 (as a result of degradation by the nonsense-mediated mRNA decay (NMD) pathway) and a higher signal of the G allele of the missense variant c.7796 A > G in exon 16 with respect to that of the A wt allele, which is consistent with the two variants being located in different alleles. Overall, all these results support the position in *trans* of the two variants.

**Table 1:** *BRCA2* biallelic genetic validation and tumor sample analysis from FA663 patient. Sanger sequencing of BRCA2 exons 10 and 16 and allelic copy number analysis by MLPA for all the samples analysed.

Tissue	Tissue type	Sample type	c.1813dup	c.7796 A > G	LOH	MLPA *BRCA2*
Blood	N	fresh	het	het		2n
Skin biopsy	N	fresh	het	het		2n
Primary right IDC	P	FFPE	het	het		2n
Primary left DCIS	P	FFPE	het	het		2n
Primary colorectal tumour	P	FFPE	het	het		2n
Supraclavicular node	M	frozen	wt	G	yes	1n
Pleural node	M	frozen	wt	G	yes	1n
Hilar node	M	frozen	wt	G	yes	1n
Hepatic node	M	frozen	wt	G	yes	1n
PDX from supraclavicular node	M	fresh	wt	G	yes	1n
PDX from hilar node	M	fresh	wt	G	yes	1n

*N* normal tissue, *P* primary tumour, *M* metastatic tissue, *het* heterozygous, *IDC* invasive ductal carcinoma, *DCIS* ductal carcinoma in situ.

### The hypomorphic missense p.(Glu2599Gly) variant displays 37–54% of HRR activity

**Fig. 4:**
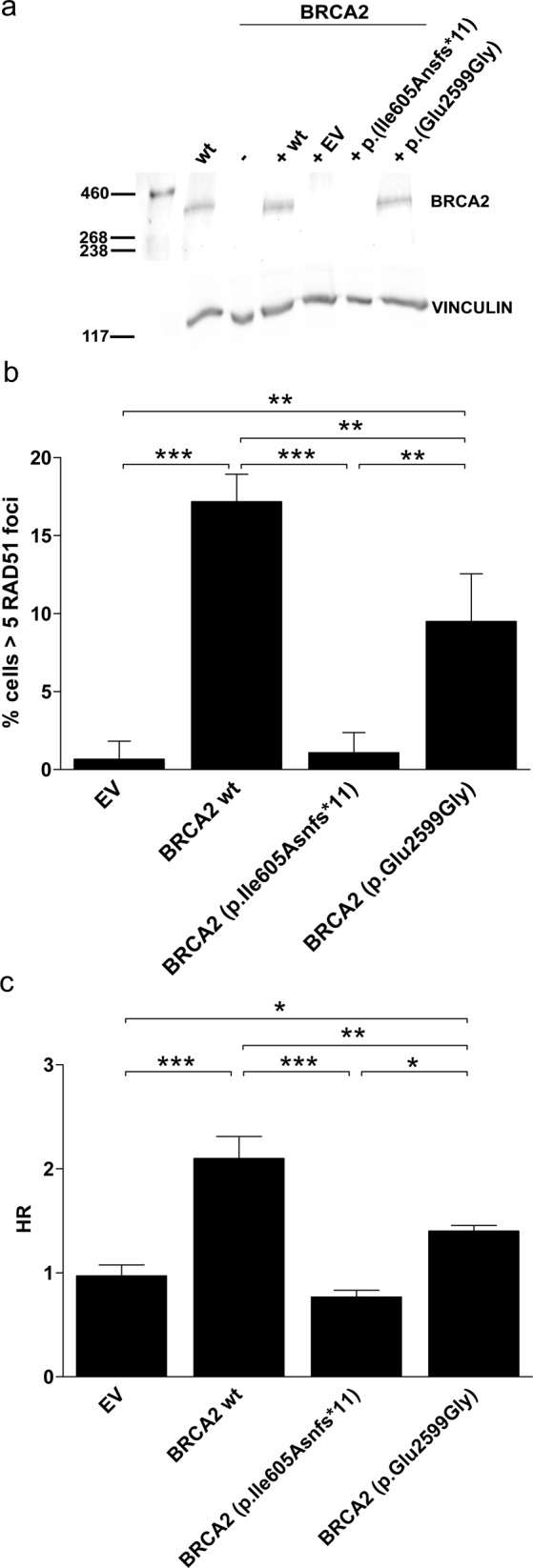
The hypomorphic missense p.(Glu2599Gly) variant has partial HRR activity. **a** Only the BRCA2 p.(Glu2599Gly) variant is expressed. Immunoblotting of BRCA2 in wt and BRCA2 KO HEK293T transiently transfected with pCDNA3 wt BRCA2, empty vector (EV), BRCA2 p.(Ile605Asnfs*11) and BRCA2 p.(Glu2599Gly), 48 h after transfection. Vinculin antibody was used as a loading control on the same sample run and processed in parallel. **b** BRCA2 p.Glu2599Gly variant shows partial complementation of RAD51 foci formation. IR-IF analysis of RAD51 in HEK293T BRCA2 KO cell line expressing wt BRCA2, EV, BRCA2 p.(Ile605Asnfs*11) and BRCA2 p.(Glu2599Gly), 6 h after 5 Gy. Mean of three independent experiments represented. Error bars show s.d. Statistics are done with the One-Way ANOVA test and Bonferroni correction **P < 0.01, ***P < 0.001. **c** Intermediate HR efficiency of *p*.(Glu2599Gly) BRCA2 expressing cells. GFP expression from the DR-GFP reporter was analysed as a measure of HR activity. The percentage of GFP-positive cells for each variant was shown. Results represent the mean of at least four independent experiments. Error bars represent s.d. Statistics are done with the One-Way ANOVA test and Newman–Keuls multiple comparison tests, *P < 0.05, **P < 0.01, ***P < 0.001.

Next, we generated a cellular system to quantify mutation-specific levels of HRR by genetic complementation of a human-derived *BRCA2* deficient cell line, stably transfected with a GFP-based construct to detect HRR (*BRCA2* KO HEK293T DR-GFP)^10^. The *BRCA2* KO cell line was generated in our laboratory by CRISPR/Cas9 technology and it was functionally validated (Figure S3). Plasmids containing wt *BRCA2* cDNA, empty vector (EV), and *BRCA2* cDNA bearing either p.(Ile605Asnfs*11) or p.(Glu2599Gly) were transiently transfected in the *BRCA2* KO HEK293T-DR-GFP. No expression of p.(Ile605Asnfs*11) BRCA2 protein could be observed by Western blot (WB) analysis, while the missense BRCA2 p.(Glu2599Gly) was expressed at comparable levels with the endogenous wt BRCA2 (Fig. 4A and Figure S4). *BRCA2* mRNA expression was detected in PDX332 (derived from the supraclavicular node) to a similar level of *BRCA2*-wt PDXs, further evidencing the expression of the missense BRCA2 p.(Glu2599Gly) variant (Figure S5). The HRR capacity of the two *BRCA2* variants was further quantified, using both an ionizing radiation-induced RAD51 foci formation (IR-IF) assay (Fig. 4B) and the DR-GFP reporter test by flow cytometry (Fig. 4C and Figure S6). With both techniques, cells expressing the missense allele had intermediate levels of HRR compared to the *BRCA2* wt reconstituted cells that exhibited functional HRR, or the cells expressing the p.(Ile605Asnfs*11) that were HRR deficient (Fig. 4B, C). We established that the residual HRR function in the missense variant p.(Glu2599Gly) was 54%± 11 (s.e.m) (Fig. 4B) and of 37% ± 7 (s.e.m) (Fig. 4C). In agreement with these findings, we observed that the level of baseline RAD51 foci in tumor samples from the metastatic supraclavicular node and the two PDXs were intermediate and significantly different to *BRCA2* wt and mutant breast tumors (Figure S7).

## Discussion

The role of *BRCA2* in DDR is relevant not only for tumor suppression, but also for organisms’ viability. Preceding studies in mice demonstrated that *Brca2* is essential for normal embryonic development^17^ and indirect evidence indicates that most of the time this is also true for humans. Pathogenic variants such as c.5946delT (also known as 6174delT) with a carrier frequency of around 1–1.5% in Ashkenazi Jewish population have not been ever detected in the homozygous state as well as many common *BRCA2* pathogenic variants that are located in exon 11^9^. Nevertheless, it has been shown that biallelic deleterious *BRCA2* variants in humans are compatible with life, despite being associated to severe forms of FA and childhood cancer predisposition^6–9^. In line with these formerly reported cases^6^, FA patients with biallelic *BRCA2* pathogenic variants previously identified in our laboratory presented elevated chromosomal fragility, severe congenital aberrations and/or high cancer incidence by age 3 or younger (Table S1). On the contrary, patient FA663 had a tumor-free childhood and mild Fanconi Anemia-associated clinical features, which remained practically unnoticed to clinicians. Our work focuses on the detailed molecular characterization of a patient being a carrier of a pathogenic variant and a hypomorphic variant in *BRCA2* that survived to adulthood with mild Fanconi Anemia-associated clinical features, but nonetheless developed young-onset breast cancer and had fatal evolution. Our functional complementation studies showed that p.(Glu2599Gly) missense allele retains between 37 and 54% of normal HRR activity levels, likely sufficient to suppress BMF, leukemia, and solid tumors during childhood, but not breast or colon cancer at adult age. Our accurate molecular screening of DNA from normal and tumor tissues demonstrated that the c.1813dup and c.7796 A > G variants were positioned in *trans*. The predicted frameshift caused by the FA663 variant c.1813dup produces a premature stop codon, which implies a degradation of the resulting transcript by NMD^18^. Our results of Sanger sequencing of blood and fibroblasts cDNA show a low signal of one allele supporting the NMD hypothesis (Figure S2).

Most of the BRCA-associated tumors undergo the loss of the BRCA-wt allele during tumor evolution, which turns the tumor into an HRR deficient (HRD) state and results in tumor sensitivity to DNA damaging chemotherapy. Intriguingly, while the primary tumors from this patient did not undergo LOH (Table 1; Fig. 3B–D), the metastatic tumors lose the *BRCA2* truncating variant, retaining the missense mutated allele (Table 1; Fig. 3E–F and Figure S1B–E). This could be an evolutionary advantage acquired by the metastatic tumors, rendering them capable of partly repairing the DNA damage induced by chemotherapy and resulting in chemoresistance. This hypothesis underscores an active role of the missense variant in the tumorigenic phenotype.

Remarkably, at least six FA cases of biallelic *BRCA2* patients due to the combination of one clearly deleterious allele and one missense allele have been previously reported^6^. Three of the variants, p.(Lys2729Asn), p.(Asn372His), and p.(Ile2490Thr) showed functional HRR^19,20^ and they appear classified in ClinVar as “Benign” (by expert review), implying that were probably not the second causal variants in these FA-D1 patient^6^. The variants, p.(Leu2510Pro) and p.(Trp2626Cys) were able to partially rescue the lethality of Brca2-null (*Brca2*^*KO/KO*^*)* ES cells, but with a 70–80% reduction in the number of colonies and an HRR severely impaired^19,20^. Cells expressing these variants had residual HRR activity, below 5%, and a 20-fold increase in ICLs induced chromosome fragility when compared to the wt BRCA2 expressing cell lines^19,20^. Similarly, the p.(Arg2336His) variant, due to the c.7007 G > A substitution in DNA, also behaves as a *BRCA2* null allele when expressed in ES cells^19,20^. The c.7007 G > A change is a splicing alteration that generates two frameshift mRNA transcripts (skipping exon 13 and exons 12–13, respectively), with the appearance of an early stop codon in exon 14^19,20^. Since functional studies demonstrated a deep impairment of HRR^20^, these last three variants cannot be considered hypomorphic, in agreement with the acute FA phenotype and early cancer predisposition shown by these patients^6^. In contrast, p.(Glu2599Gly) found in our patient exhibited substantial levels of HRR in all functional assays performed in our study but well below the wt levels (Fig. 2, Fig. 4 and Figure S5), in accordance to recent data^21^. Moreover, we observed the same intermediate chromosome fragility in both primary lymphocytes and fibroblasts from FA663 patients. This is indicative of a hypomorphic effect of the p.(Glu2599Gly) variant and not of FA somatic mosaicism. Indeed, a diagnosis of FA somatic mosaicism is usually consistent with the presence of higher induced chromosome fragility in patient’s fibroblasts than patient’s lymphocytes^12,22,23^.

The p.(Glu2599Gly) variant presented by our patient is located in the helical domain (2479 to 2668aa) of the DNA binding region of BRCA2. This domain interacts with DSS1^24^, which promotes the exchange of BRCA2-RPA1 to BRCA2-RAD51 and thus the ssDNA interaction^25^. The BRCA2 DNA binding domain is also involved in the stabilization of BRCA2 and in the regulation of R-loop-associated DNA damage, a particular source of damage resolved by FA proteins^26,27^. In addition, it has been shown that hypomorphic missense variants in the DNA binding domain of BRCA2 with partial effects on protein function may confer moderate risks of breast cancer^28^, being the hypomorphic variants associated with this domain the only missense variants in BRCA2 increasing cancer risk. Although we show that *BRCA2* p.(Glu2599Gly) is hypomorphic in terms of DNA repair function, this study cannot provide a quantification of its estimated reduced cancer penetrance, due to the fact that it is presented together with another clearly deleterious variant and the lack of a significant number of patients harboring this missense variant.

Our findings together with two family study cases that reported biallelic *BRCA2* patients with a less severe FA phenotype and early onset breast and/or colon cancer susceptibility^29,30^ are relevant for the interpretation of variants with unknown significance (VUS) in *BRCA2*. In the evaluation of the potential pathogenicity of a variant in *BRCA2*, it is considered that its co-occurrence in *trans* with a clearly pathogenic variant indicates its benign character^31^. However, our results show that this criterion cannot be applied without careful exploration of the clinical phenotype.

We conclude that the *BRCA2* p.(Glu2599Gly) is a truly hypomorphic *BRCA2* allele that retains sufficient HRR activity for normal organism development and to minimize FA-related phenotypes, but not enough to avoid early onset of tumorigenesis and severe chemotoxicity. Furthermore, we propose a straightforward methodology that produces conclusive results in a reasonable turnaround time to classify *BRCA2* variants of uncertain significance (VUS) in cancer patients.

## Methods

### Cell lines and cultures

Blood cultures were established from patient FA663: 10% of blood (in heparin) and 90% RPMI (Gibco) with 15% FBS, 1% antibiotics, 1% L-Glutamine, and 1% phytohemaglutinin. Wild type primary fibroblasts were previously established from a 4-year-old patient undergoing phimosis surgery; homozygous *BRCA2* c.469 A > T, p.Lys157* and FA663 primary fibroblasts were established from skin biopsies of patients. Cells were grown in DMEM with 20% FBS (Biowest) and antibiotics. Human cell lines HEK293T (Human Embryonic Kidney 293 T cells, ATCC CRL–11268) were cultured in DMEM 10% FBS and antibiotics.

### Chromosome fragility assays and G2/M cell cycle arrest

For the Chromosome fragility test, peripheral blood T-lymphocytes and primary fibroblasts were treated with diepoxybutane (DEB) at the indicated concentrations and processed as in^11,32^. For the G2/M cell cycle arrest analysis, primary fibroblasts were treated with Mitomycin-C at the indicated concentrations and processed as in^32^.

### RAD51 foci formation

75000 fibroblast cells were grown on 4-well Millicell EZ slide units (Merck Millipore) and irradiated with 5 Gy. Cells were fixed with 4% formaldehyde in PBS (Quimigen), incubated in Triton X-100 0.5%, and blocked with 10% BSA (Sigma) TPBS. Slides were incubated with RAD51 antibody (H-92, 8349 Santa Cruz) diluted 1:500 overnight at 4 °C, washed and incubated for 30 min at 37 °C with anti-rabbit Alexa 568 conjugated secondary antibody (Molecular Probes) diluted 1:500. Coverslips were mounted in Vectashield Mounting Medium (H-1000, Vector Laboratories, Inc.), containing DAPI. 300000 HEK293T cells were seeded on coverslips (previously coated with Poly-L-Lysine) in 6 cm Petri dishes and the same procedure was followed.

### RAD51 on formalin-fixed paraffin-embedded (FFPE) samples

The homologous recombination repair activity was assessed on FFPE tumor samples as previously described^33,34^. In brief, γH2AX and RAD51 foci were determined in cells in the S/G2-phase of the cell cycle (geminin-positive). The following primary antibodies were diluted in DAKO Antibody Diluent (K8006) and incubated at room temperature for 1 h: rabbit anti-RAD51 (Abcam ab133534, 1:1000), mouse anti-geminin (NovoCastra NCL-L, 1:100 in PDX samples, 1:60 in patient samples), rabbit anti-geminin (ProteinTech 10802-1-AP, 1:400) and mouse anti-phospho-H2AX (Millipore #05-636, 1:200). Secondary antibodies were diluted in blocking buffer and incubated for 30 min: goat anti-rabbit IgG Alexa fluor 568 (Invitrogen A11011; 1:500), goat anti-mouse IgG Alexa fluor 488 (Invitrogen A11001; 1:500), donkey anti-mouse IgG Alexa fluor 568 (Invitrogen A10037; 1:500), and goat anti-rabbit IgG Alexa fluor 488 (Invitrogen A11008; 1:500). Finally, sections were dehydrated and mounted with DAPI ProLong Gold antifade reagent (ThermoFisher P36941). Scoring was performed blindly using a 60×-immersion oil objective with a Nikon Eclipse Ti-E microscope. The amount of dsDNA damage served as a quality check and was quantified by scoring the percentage of geminin-positive cells with γ-H2AX foci. The RAD51 score was obtained by quantifying the percentage of geminin-positive cells with 5 or more nuclear foci. A pre-defined cut-off of 10% was used for RAD51 to differentiate between homologous recombination deficient (HRD) and proficient (HRP) tumors^33,34^.

### PARPi survival assays

1000 fibroblast cells (wt, BRCA2 and FA663) were seeded on 96-well plates and treated with 0, 0.5, 5, 50, 500, and 5000 nM Olaparib (Selleckchem) or 0, 50, 500, 5000, and 50000 nM Veliparib (Deltaclon S.L.). Cells were fixed adding 10% trichloroacetic (TCA) (Sigma) solution, stained with 0.4% Sulforhodamine B (Sigma) acetic acid, and resuspended with 10 mM Tris (Sigma) solution. OD has measured 510 nm with a microplate reader (Multiskan Sky Touch Drop plate, ref51119600DP).

### BRCA2 mutant cell line generation

The selected plasmid to generate CRISPRs constructs was pX330 (pX330 – U6 – Chimeric_BB-CBh-HSpCas9, Addgene 42230)^35^. Selected sequence to target *BRCA2* is GTTGATTTCCAGTACCAACT, located in exon 11 and previously described^35^. Primers used for CRISPR pX330 plasmid generation are forward 5’-caccgttgatttccagtaccaact and reverse 5’-aaacagttggtactggaaatcaac. Three days after, cells were analyzed by flow cytometry using the FACSAria II (BD Biosciences) and RFP+ GFP+ cells were sorted. Clones were analyzed by WB and genotyped with forward 5’-tcagactgcaagtgggaaaa-3’ and reverse 5’-ggtctttacaggcctctctgt-3’ primers.

### BRCA2 plasmids

Human *BRCA2* cDNA (NM_000059) was cloned in the pcDNA3 vector (Invitrogen). The two *BRCA2* deleterious variants were introduced in the pcDNA3-BRCA2 plasmid using the QuikChange II XL Site-Directed Mutagenesis Kit (Agilent) using for p.(Ile605Asnfs*11) variant forward 5’-ctatacatgatgaaacatcttataaaggaaaaaaaataaccgaaagaccaaaaatca and reverse 5’-tgatttttggtctttcggttattttttttcctttataagatgtttcatcatgtatag primers and for p.(Glu2599Gly) variant forward 5’-acacagagccctataaaatccttcttttccagcctttcc- and reverse 5’-ggaaaggctggaaaagaaggattttatagggctctgtgt primers.

### Plasmid transfection

Transfections were carried out using 10 μL Lipofectamine 2000 (Life Technologies) and 250 μL Optimem (Life Technologies) plus 10 μg of each pCDNA3 plasmid construct the day after plating 600000 HEK293T cells/well in 6-well plates with complete medium.

### Western blot antibodies

Primary antibodies used were Rabbit anti-BRCA2 (Ab123491, Abcam) and mouse anti-vinculin (Ab18058, Abcam) were diluted 1:1000 and 1:2500, respectively; and secondary antibodies, goat anti-rabbit of (Bethyl Laboratories, Inc.) and goat anti-mouse (Santa Cruz), both diluted 1:2000.

### HRR assay

HRR assay was performed as in^36^ with some modifications: 200000 cells HEK293T expressing pDR-GFP construct in a stable manner were seeded in 12-well plates. pCDNA3 plasmids were co-expressed with an I-SceI expressing plasmid by transient transfection. HR-dependent repair of I-SceI induced DNA double-strand breaks was quantified by fluorescence-activated cell sorting (FACS) of GFP + cells after 48 h.

### Generation of PDX models

Biopsy specimens from metastatic lesions were immediately implanted into the lower flank of 5-week-old female NOD SCID GAMMA mice (NSG, Charles River) and then expanded in NMRI-Foxn1nu/nu mice (NMRI, Janvier Labs). This procedure was conducted following the European Union’s animal care directive (2010/63/EU) and was approved by the Ethical Committee of Animal Experimentation of the Vall d’Hebron Research Institute.

### BRCA2 mRNA expression

RNA was extracted from PDX samples (15–30 mg) by using the PerfectPure RNA Tissue kit (five Prime). cDNA was obtained using the PrimeScript RT Reagent kit (Takara). Quantitative RT-qPCR was performed in a 7900HT Fast Real-Time PCR System (Applied Biosystems) using TaqMan Universal Master Mix II (Applied Biosystems) and predesigned human-specific primers and TaqMan probes (Hs99999908 for GUSB, Hs99999903 for ACTB, and Hs01037414 for *BRCA2*). The comparative CT method was used for data analysis, in which geNorm algorithms were applied to select the most stably expressed housekeeping genes (GUSB and ACTB) and geometric means were calculated to obtain normalized CT values^37^.

### In silico prediction tools

The in silico prediction of functional impact of the missense *BRCA2* c.7796 A > G variant was performed using BRASS (a specific tool for *BRCA1* and *BRCA2* genes; https://www.biotoclin.org/BRASS#top)^38^ and Alamut software v2.10 (Interactive Biosoftware) that includes three protein-function prediction tools: Align-GVGD, (http://agvgd.hci.utah.edu/agvgd_input.php), SIFT (https://sift.bii.a-star.edu.sg/), and PolyPhen-2 (http://genetics.bwh.harvard.edu/pph2/).

### DNA extraction and genetic variant screening

Genomic DNA was extracted from patient peripheral blood and skin biopsy samples using Puregene Genome DNA Purification Kit (Gentra System). DNA from tumor FFPE samples was extracted either with Maxwell^®^16 FFPE Plus LEV DNA Purification Kit (Promega Corp.) or QIAamp DNA FFPE Tissue Kit (QIAGEN). DNA from fresh or frozen tissues was isolated using DNeasy blood and tissue kit (QIAGEN). Exome sequencing procedure was performed as described in^13^.

The *BRCA2* variants c.1813dup (exon 10; forward 5’-gcctctgaaagtggactgga-3’ and reverse 5’-aaaaacacagaaggaatcgtca-3’) and c.7796 A > G (exon 16; forward 5’-gtgtgatacatgtttactttaaattg-3’ and reverse 5’-gttcgagagacagttaagagaag-3’) were genotyped by PCR of genomic DNA and Sanger sequencing. PCR products were purified using ExoSAP-IT PCR Product Cleanup Reagent (ThermoFisher), sequenced using BigDye Terminator v3.1 Cycle Sequencing kit (Applied Biosystems) and run in a Genetic Analyzer ABI3130xl (Applied Biosystems). Reference transcript *BRCA2* NM_000059.3 was used for sequence alignment and variant annotation.

### RNA extraction, reverse transcription and cDNA amplification

RNA was isolated from patient’s peripheral blood samples and cultured fibroblasts using Trizol Isolation Reagent (ThermoFisher Scientific). RNA was cleaned up using Rneasy Minikit (QIAGEN) and digested with RNase-Free DNase Set (QIAGEN) to remove genomic DNA. A total of 500 ng were reverse transcribed with PrimeScript RT reagent kit (Takara) that supplies random and oligo-dT primers. *BRCA2* cDNA was amplified in two PCR fragments spanning exons 10 to 14 (forward 5’-tatgtccaaatttaattgataat-3’ and reverse 5’-ttggtctgcctgtagtaatc-3’) and 11 to 18 (forward 5’-ttctgatgttcctgtgaaaacaa-3’ and reverse 5’-attctggggcttcaagaggt-3’), respectively, using Expand™ Long Template PCR System (Roche). Sequencing was performed with BigDye Terminator v3.1 Cycle Sequencing kit (Applied Biosystems) in a Genetic Analyzer ABI 3130xl (Applied Biosystems). Sanger electropherograms were visualized using SeqScape v2.6 and Sequencing Analysis v2.6 softwares (Applied Biosystems).

### DNA copy number analysis by multiplex ligation-dependent probe assay (MLPA)

Allelic copy number changes in *BRCA2* were performed by MLPA kit #P090 (MRC-Holland), and resulting electrophoresis on an ABI 3130xl capillary sequencer (Applied Biosystems). Analysis was done using Coffalyser.Net software (MRC-Holland, Amsterdam, The Netherlands). Blood samples from healthy donors were included for sample normalization.

### Ethics approval

Patient blood and samples collection was carried out under patient consent and the positive approval of the Clinical Research Ethics Committee of Vall d’Hebron Hospital (Barcelona, Spain), project number PR(AG)64/2007. Fresh primary or metastatic tumor samples were collected prospectively for implantation into nude mice at VHIO under an institutional review board (IRB)-approved protocol and the associated informed consent, project number PR(AG)173-2015. The experiments conformed to the principles of the WMA Declaration of Helsinki and the Department of Health and Human Services Belmont Report.

Animal experiments were conducted following the European Union’s animal care directive (2010/63/EU) and the protocols were approved by the Ethical Committee of Animal Experimentation of the Vall d’Hebron Research Institute and the appropriate governmental agency and carried out in accordance with the approved guidelines.

### Consent to participate

Written informed consent was obtained from the patient for publication of this case report. The Clinical Research Ethics Committee of Vall d’Hebron Hospital (Barcelona, Spain) approved the study.

### Reporting summary

Further information on research design is available in the Nature Research Reporting Summary linked to this article.

## Supplementary information

Supplementary information

Reporting Summary

## Data Availability

The datasets generated and/or analysed during the current study are available from the corresponding author on reasonable request.
