# Supplementary material for: Clinical consequences of BRCA2 hypomorphism
Source: NPJ Breast Cancer. 2021 Sep 9;7:117. doi: 10.1038/s41523-021-00322-9 (PMC8429460; doi:10.1038/s41523-021-00322-9)
Supplement: Supplementary file 1 — Supplementary information [file 41523_2021_322_MOESM1_ESM.pdf]

SUPPLEMENTARY TABLES

Supplementary Table 1. Biallelic BRCA2 patients' description.

Patient	% aberrant cells		Aberrations/ Aberrant cell		CFI	Mutation 1	Mutation 2	Age		*Exitus* cause	Phenotype
	-DEB	+DEB	-DEB	+DEB				Diagnosis	*Exitus*		
FA62	64.0	100.0	2.5	9.0	900	15-16 exons del<br>No protein	1597del<br>p.(Thr533Leufs*25)	3m	<1y	Wilms' tumor	typical FA-like face, multiple congenital abnormalities in several organs (including heart), short stature, Wilm's tumor (with metastasis), multiple cancer family history
FA158	8.0	38.0	1.5	2.9	143	8488-1G>A<br>p.(Trp2830_ Lys2833del)	8488-1G>A<br>p.(Trp2830_ Lys2833del)	2y	8y	Sudden death	multiple congenital abnormalities, severe prenatal growth delay, psychomotor delay, microcephaly, severe hypotonia, scoliotic column, cardiac alterations, minor abnormalities in hands and feet, West syndrome, anal atresia, bilateral radial axis hypoplasia, peculiar facies, non-medullary aplasia, 11 bilateral ribs
FA548	12.0	74.0	2.3	5.8	545	658_659delGT<br>p.(Val220Ilefs*4)	7006delC<br>p.(Arg2336Alafs*31)	1y	2y	Wilms' tumor	anemia, thrombopenia, ectopic kidney, hyperpigmentation, weight alterations, Wilm's tumor, kidney neuroblastoma
FA663	16.0	32.0	1.5	2.9	119	c7796A>G<br>p.(Glu2599Gly)	1813dup<br>p.(Ile605Ansfs*11)	33y	35y	Breast cancer	low birth weight, probable learning disorder, skin hyperpigmentation, right hypochondria hypopigmentation, hiatal hernia, hepatic steatosis, herniated disc, Breast Cancer DIC (Ductal Infiltrating Carcinoma)
Negative control	3.2	4.9	1.2	3.1	18*						

Chromosomal Fragility Index (CFI) (% aberrant cells x Aberrations/ Aberrant cells) *Threshold value is 40(Castella et al., 2011)

SUPPLEMENTARY FIGURES

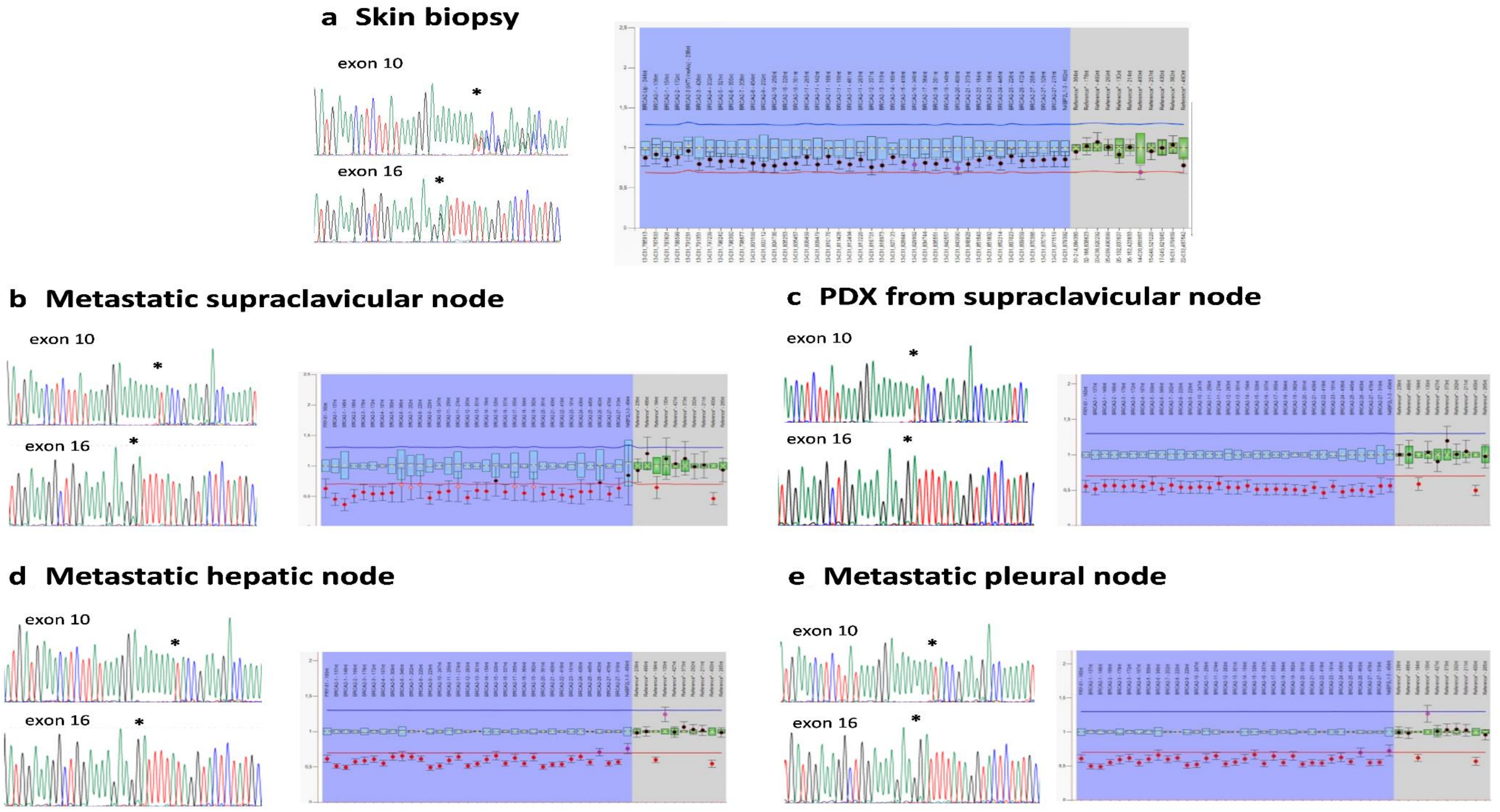

Supplementary Figure 1. Sanger sequences and MLPA results of skin biopsy, metastatic tumor and PDXs samples from the biallelic *BRCA2* patient. Skin biopsy shows the two *BRCA2* pathogenic variants in heterozygosis and two copies of *BRCA2*, while all tumor related samples show loss of heterozygosis (LOH) and one allele of *BRCA2*.

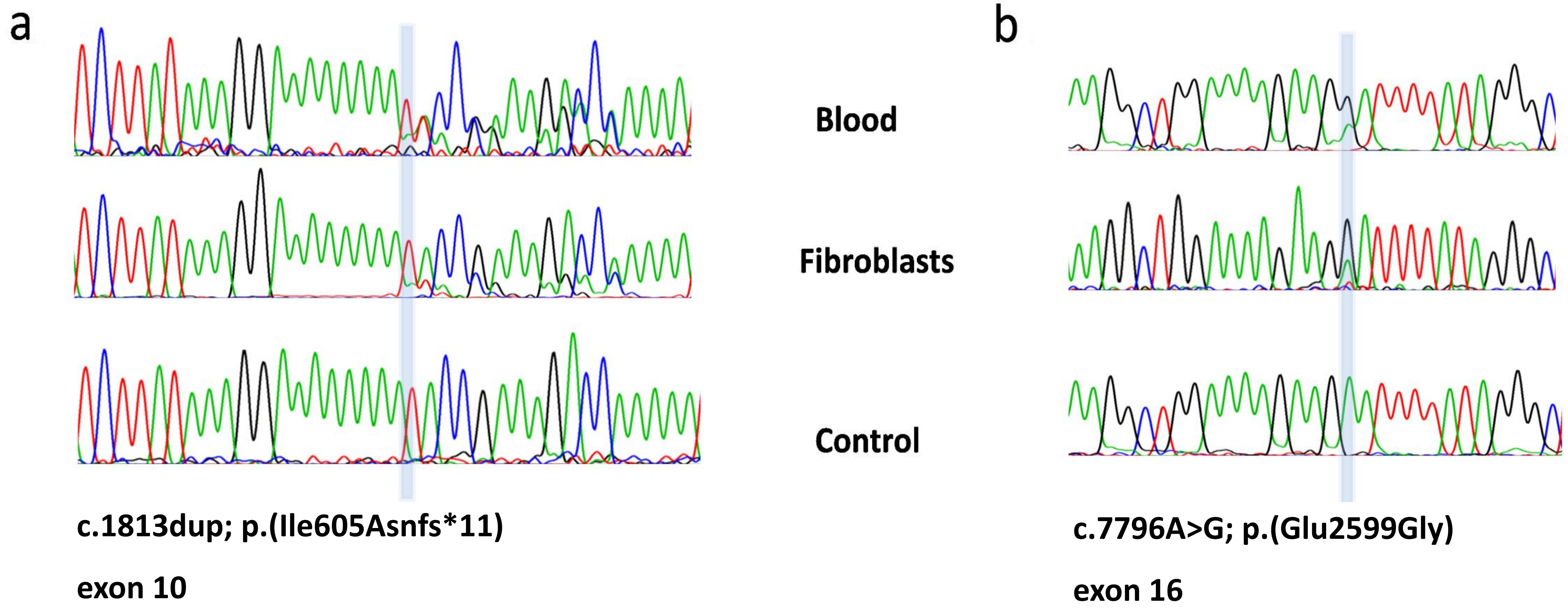

Supplementary Figure 2. cDNA Sanger sequencing on blood cells and fibroblasts cultured from a skin biopsy sample.

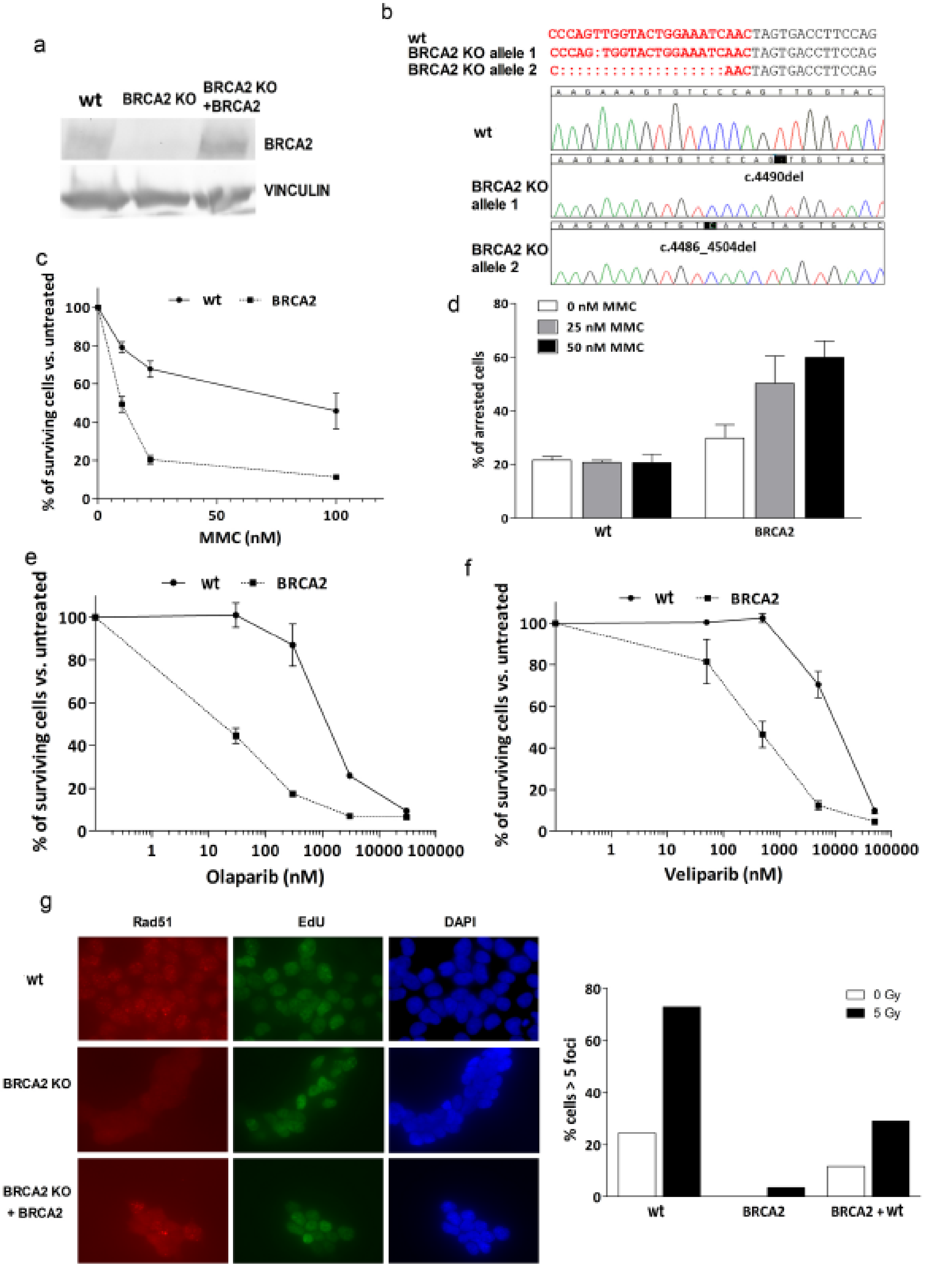

Supplementary Figure 3. BRCA2 CRISPR/Cas HEK923T cell line generation. a WB analysis of BRCA2 in wt, BRCA2 KO and BRCA2 KO +BRCA2. **b** BRCA2 null cells possess two frameshift mutations in heterozygosis. **c** MMC sensitivity. **d** cell cycle arrest enhancement. **e** Olaparib and **f** Veliparib sensitivity. **g** RAD51 immunostaining in response to irradiation (5 Gy).

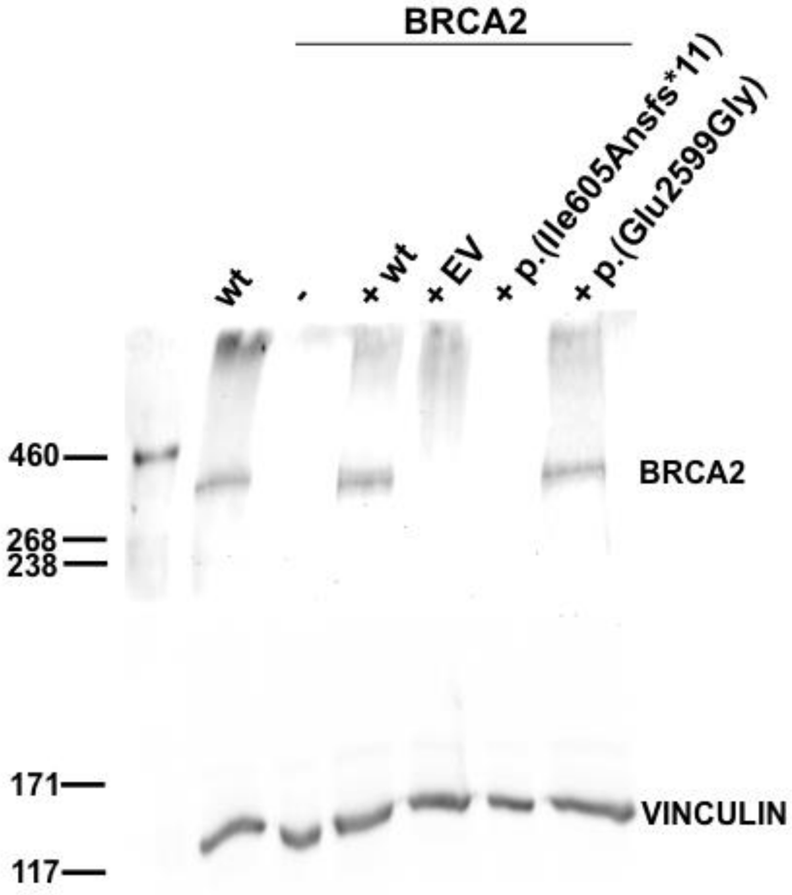

Supplementary Figure 4. Full western blot image corresponding to Figure 4A.

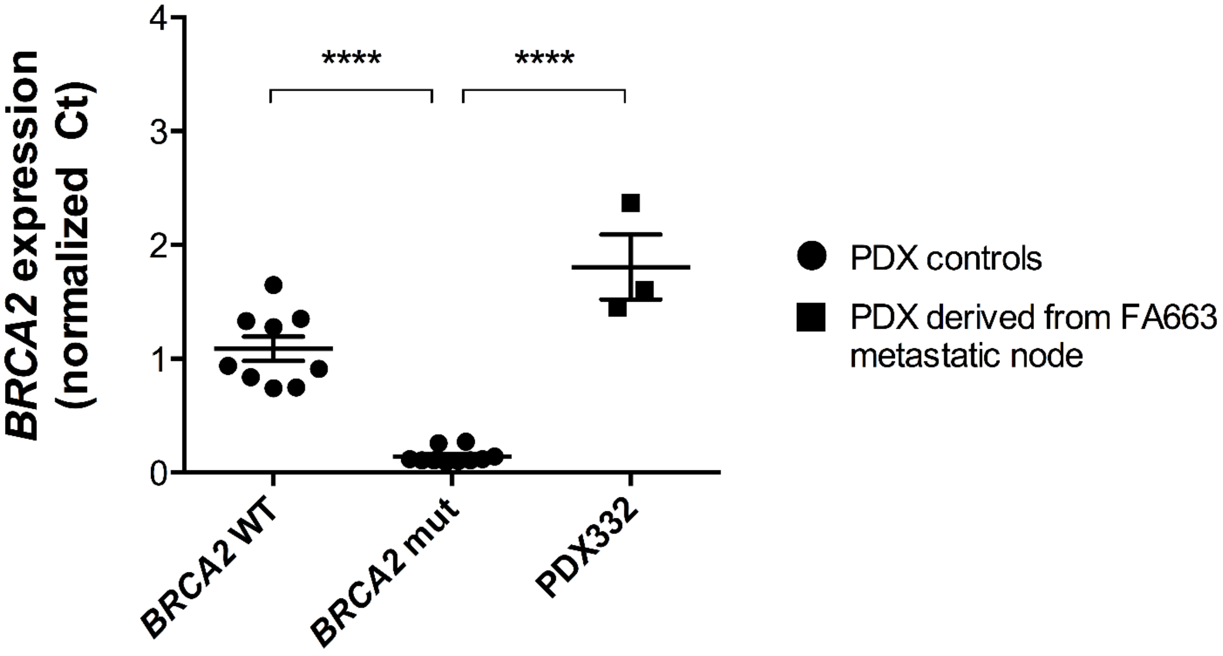

Supplementary figure 5. *BRCA2* mRNA expression measured by RT-qPCR in one of the PDX models, derived from one of the patient metastatic nodes (the supraclavicular) with LOH retaining only *BRCA2* c.7796A>G allele. Three biological replicates of PDX332 are shown. A pool of *BRCA2* wild-type and BRCA2 mutated PDX models were used as controls. Error bars represent SEM of biological replicates. Statistics are done with the One-Way ANOVA test and Bonferroni correction, *P<0.05.

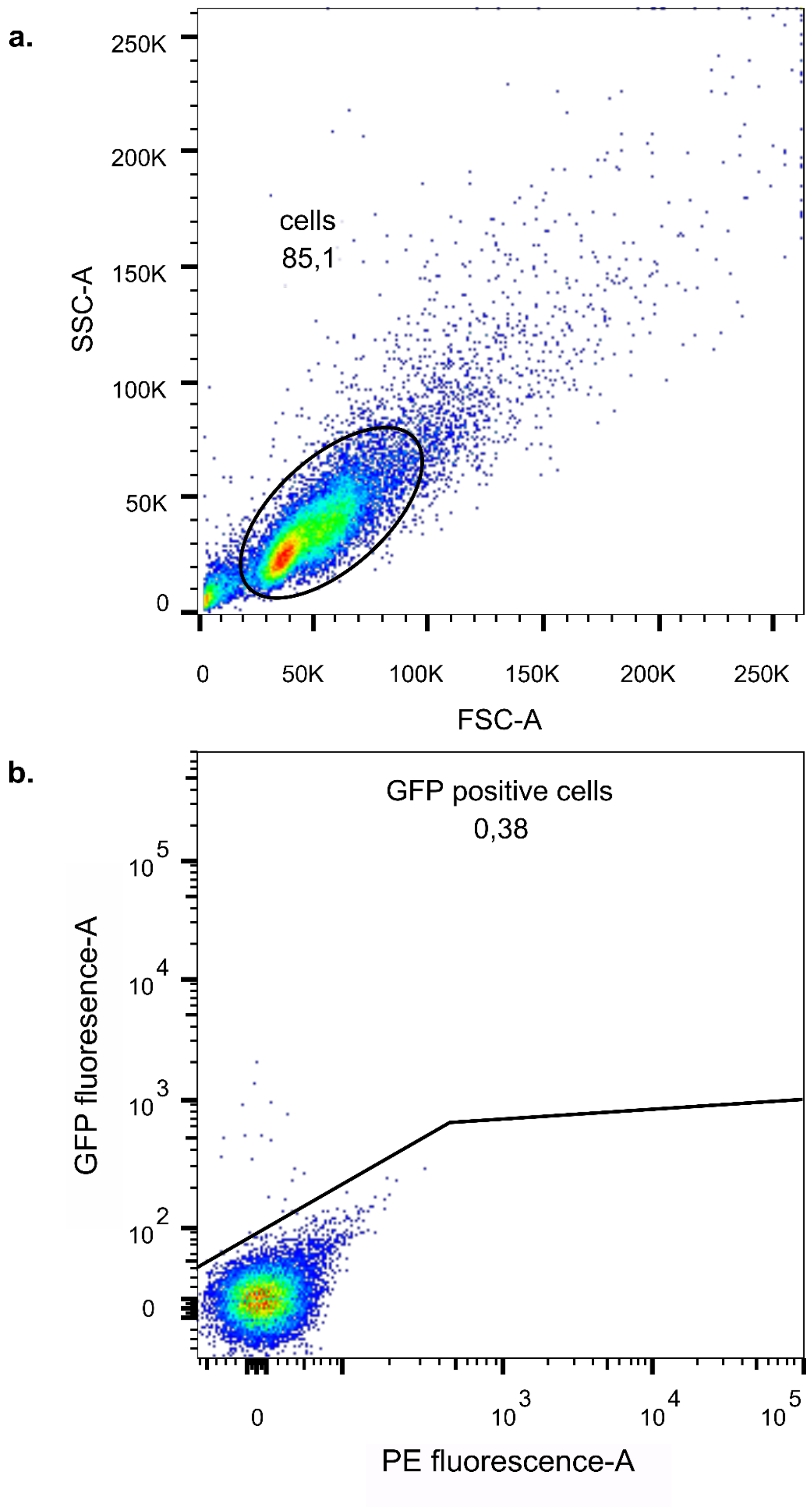

Supplementary Figure 6. Flow cytometry gating strategy to identify GFP cellular fraction for HR assay. **a**, SSC-A x FSC-A gating to obtain the general cellular population. **b**, GFP-fluorescence-A x PE fluorescence-A gating to discriminate between GFP-fluorescent cellular fraction and non-fluorescent cellular fraction, gates corresponding to Figure 4C.

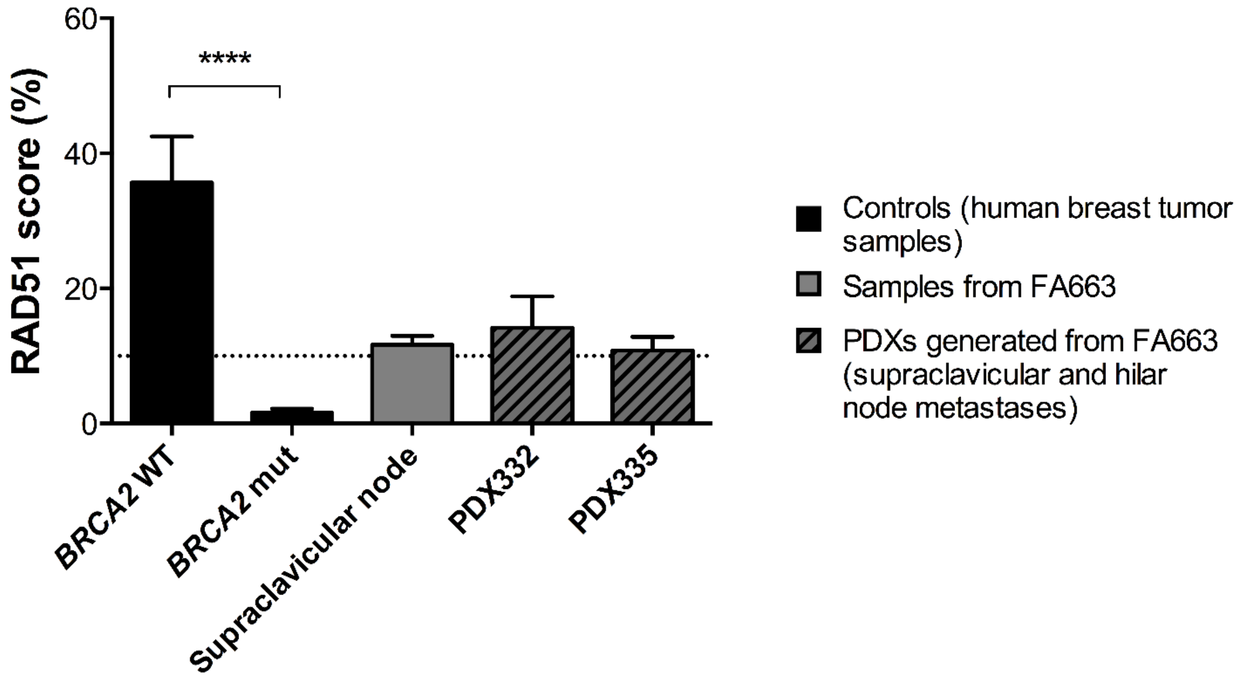

Supplementary Figure 7. **Intermediate levels of RAD51 foci in FFPE metastatic tumor and in patient-derived xenograft (PDX) tumor model from two metastatic nodes that showed LOH retaining exclusively the *BRCA2* c.7796A>G allele.** FFPE breast tumors from *BRCA2* non-carriers (*BRCA2* WT) or germline *BRCA2* carriers (*BRCA2* mut) were used as positive and negative controls, respectively. The dashed line indicates the threshold that discriminates between homologous recombination deficient (HRD) and proficient (HRP) tumors(31). Error bars represent SEM of biological replicates. Statistics are done with the One-Way ANOVA test and Bonferroni correction, *P<0.05.
