# Supplementary material for: Clinical consequences of BRCA2 hypomorphism
Source: NPJ Breast Cancer. 2021 Sep 9;7:117. doi: 10.1038/s41523-021-00322-9 (PMC8429460; doi:10.1038/s41523-021-00322-9)
Supplement: Supplementary file 2 — Reporting Summary [file 41523_2021_322_MOESM2_ESM.pdf]

nature research

Corresponding author(s): Balmaña and Surrallés

Last updated by author(s): Jan 15, 2021

Reporting Summary

Nature Research wishes to improve the reproducibility of the work that we publish. This form provides structure for consistency and transparency in reporting. For further information on Nature Research policies, see our Editorial Policies and the Editorial Policy Checklist.

Statistics

For all statistical analyses, confirm that the following items are present in the figure legend, table legend, main text, or Methods section.

n/a	Confirmed	
☐	☒	The exact sample size (*n*) for each experimental group/condition, given as a discrete number and unit of measurement
☐	☒	A statement on whether measurements were taken from distinct samples or whether the same sample was measured repeatedly
☐	☒	The statistical test(s) used AND whether they are one- or two-sided<br>*Only common tests should be described solely by name; describe more complex techniques in the Methods section.*
☒	☐	A description of all covariates tested
☐	☒	A description of any assumptions or corrections, such as tests of normality and adjustment for multiple comparisons
☐	☒	A full description of the statistical parameters including central tendency (e.g. means) or other basic estimates (e.g. regression coefficient) AND variation (e.g. standard deviation) or associated estimates of uncertainty (e.g. confidence intervals)
☒	☐	For null hypothesis testing, the test statistic (e.g. *F*, *t*, *r*) with confidence intervals, effect sizes, degrees of freedom and *P* value noted<br>*Give P values as exact values whenever suitable.*
☒	☐	For Bayesian analysis, information on the choice of priors and Markov chain Monte Carlo settings
☒	☐	For hierarchical and complex designs, identification of the appropriate level for tests and full reporting of outcomes
☒	☐	Estimates of effect sizes (e.g. Cohen's *d*, Pearson's *r*), indicating how they were calculated

Our web collection on statistics for biologists contains articles on many of the points above.

Software and code

Policy information about availability of computer code

Data collection	*Provide a description of all commercial, open source and custom code used to collect the data in this study, specifying the version used OR state that no software was used.*
Data analysis	*Provide a description of all commercial, open source and custom code used to analyse the data in this study, specifying the version used OR state that no software was used.*

For manuscripts utilizing custom algorithms or software that are central to the research but not yet described in published literature, software must be made available to editors and reviewers. We strongly encourage code deposition in a community repository (e.g. GitHub). See the Nature Research guidelines for submitting code & software for further information.

Data

Policy information about availability of data

All manuscripts must include a data availability statement. This statement should provide the following information, where applicable:

- Accession codes, unique identifiers, or web links for publicly available datasets
- A list of figures that have associated raw data
- A description of any restrictions on data availability

The datasets generated and or analysed during the current study are avaible from the corresponding author on reasonable request.

Field-specific reporting

Please select the one below that is the best fit for your research. If you are not sure, read the appropriate sections before making your selection.

☒ Life sciences ☐ Behavioural & social sciences ☐ Ecological, evolutionary & environmental sciences

For a reference copy of the document with all sections, see nature.com/documents/nr-reporting-summary-flat.pdf

Life sciences study design

All studies must disclose on these points even when the disclosure is negative.

Sample size	Standard protocols were followed according to each reference.
Data exclusions	*Describe any data exclusions. If no data were excluded from the analyses, state so OR if data were excluded, describe the exclusions and the rationale behind them, indicating whether exclusion criteria were pre-established.*
Replication	From three to five replicated experiments were performed to reduce data variability.
Randomization	Each sample was allocated according to their cell line procedence.
Blinding	For sample analysis, when possible, all samples were blinded.

Reporting for specific materials, systems and methods

We require information from authors about some types of materials, experimental systems and methods used in many studies. Here, indicate whether each material, system or method listed is relevant to your study. If you are not sure if a list item applies to your research, read the appropriate section before selecting a response.

Materials & experimental systems

n/a	Involved in the study
☐	☒ Antibodies
☐	☒ Eukaryotic cell lines
☒	☐ Palaeontology and archaeology
☐	☒ Animals and other organisms
☐	☒ Human research participants
☐	☒ Clinical data
☒	☐ Dual use research of concern

Methods

n/a	Involved in the study
☒	☐ ChIP-seq
☐	☒ Flow cytometry
☒	☐ MRI-based neuroimaging

Antibodies

Antibodies used	anti-RAD51 antibody (H-92, Santa Cruz), anti-rabbit Alexa 568 conjugated secondary antibody (Molecular Probes), rabbit anti-RAD51 from Abcam ab133534, mouse anti-geminin from NovoCastra NCL-L, rabbit anti-geminin from ProteinTech 10802-1-AP and mouse anti-phospho-histone H2AX from Millipore #05-636, anti-BRCA2 (Ab123491, Abcam), mouse anti-vinculin (Ab18058, Abcam), goat anti-rabbit of (Bethyl Laboratories, Inc.) and goat anti-mouse (Santa Cruz).
Validation	Anti-BRCA2 (Ab123491, Abcam) was validated using a KO cell line.

Eukaryotic cell lines

Policy information about cell lines

Cell line source(s)	Wild type primary fibroblasts were previously established from a 4 year old patient undergoing phimosis surgery from Celltec; homozygous BRCA2 c.469A>T, p.Lys157* and FA663 primary fibroblasts were established from skin biopsies of patients. Human HEK293T cell line from ATCC (ref. CRL-11268).
Authentication	Comercial cell lines were authenticated by de company.
Mycoplasma contamination	All cell lines used were tested negative for mycoplasma.
Commonly misidentified lines (See ICLAC register)	*Name any commonly misidentified cell lines used in the study and provide a rationale for their use.*

Animals and other organisms

Policy information about studies involving animals; ARRIVE guidelines recommended for reporting animal research

Laboratory animals	Biopsy specimens from metastatic lesions were immediately implanted into the lower flank of 5-week-old female NOD SCID GAMMA mice (NSG, Charles River) and then expanded in NMRI-Foxn1nu/nu mice (NMRI, Janvier Labs).
Wild animals	*Provide details on animals observed in or captured in the field; report species, sex and age where possible. Describe how animals were caught and transported and what happened to captive animals after the study (if killed, explain why and describe method; if released, say where and when) OR state that the study did not involve wild animals.*
Field-collected samples	*For laboratory work with field-collected samples, describe all relevant parameters such as housing, maintenance, temperature, photoperiod and end-of-experiment protocol OR state that the study did not involve samples collected from the field.*
Ethics oversight	Animal experiments were conducted following the European Union's animal care directive (2010/63/EU) and the protocols were approved by the Ethical Committee of Animal Experimentation of the Vall d'Hebron Research Institute and the appropriate governmental agency and carried out in accordance with the approved guidelines.

Note that full information on the approval of the study protocol must also be provided in the manuscript.

Human research participants

Policy information about studies involving human research participants

Population characteristics	n/a
Recruitment	n/a
Ethics oversight	Patient blood and samples collection was carried out under patient consent and the positive approval of the Clinical Research Ethics Committee of Vall d'Hebron Hospital (Barcelona, Spain), project number PR(AG)64/2007.

Note that full information on the approval of the study protocol must also be provided in the manuscript.

Clinical data

Policy information about clinical studies

All manuscripts should comply with the ICMJE guidelines for publication of clinical research and a completed CONSORT checklist must be included with all submissions.

Clinical trial registration	*Provide the trial registration number from ClinicalTrials.gov or an equivalent agency.*
Study protocol	*Note where the full trial protocol can be accessed OR if not available, explain why.*
Data collection	*Describe the settings and locales of data collection, noting the time periods of recruitment and data collection.*
Outcomes	*Describe how you pre-defined primary and secondary outcome measures and how you assessed these measures.*

Flow Cytometry

Plots

Confirm that:

- [ ] The axis labels state the marker and fluorochrome used (e.g. CD4-FITC).
- [ ] The axis scales are clearly visible. Include numbers along axes only for bottom left plot of group (a 'group' is an analysis of identical markers).
- [ ] All plots are contour plots with outliers or pseudocolor plots.
- [ ] A numerical value for number of cells or percentage (with statistics) is provided.

Methodology

Sample preparation	The HR assay was performed in HEK293T by transient transfection of the pDR-GFP plasmid. 2 days after, cells were trypsinized and resuspended in a buffer containing sodium citrate (3.4 mM), Triton (0.1%), RNase (200 µg/ml), and FBS (20%). The number of green fluorescent cells was counted in a flow cytometer. Cells were finally stained with propidium iodide (50 µg/ml), and the cell cycle distribution was determined by flow cytometry. The level of HR was quantified as the proportion of green fluorescent cells corrected by transfection rate and size of S phase. Plasmids forthe I-SceI–induced HR assay were provided by Maria Jasin, Memorial Sloan Kettering Cancer Center, New York, New York, USA.
Instrument	Calibur citometer
Software	Flowjo

Cell population abundance | *Describe the abundance of the relevant cell populations within post-sort fractions, providing details on the purity of the samples and how it was determined.*

Gating strategy | Population of interest was selected by FCS/SSC, afterwards the green fluorescence is chosen and the extracted population above 40 UF selected.

☐ Tick this box to confirm that a figure exemplifying the gating strategy is provided in the Supplementary Information.
